# The fourth national tuberculosis prevalence survey in Myanmar

**DOI:** 10.1371/journal.pgph.0000588

**Published:** 2022-06-14

**Authors:** Si Thu Aung, Wint Wint Nyunt, Myat Myat Moe, Htin Lin Aung, Thandar Lwin

**Affiliations:** 1 Department of Public Health, Ministry of Health and Sports, Naypyitaw, Myanmar; 2 National Tuberculosis Reference Laboratory, National Tuberculosis Programme, Ministry of Health and Sports, Yangon, Myanmar; 3 National Tuberculosis Programme, Ministry of Health and Sports, Naypyitaw, Myanmar; 4 Department of Microbiology and Immunology, University of Otago, Dunedin, New Zealand; University of Bath, UNITED KINGDOM

## Abstract

Tuberculosis (TB) remains a significant cause of morbidity and mortality in Myanmar. The fourth National TB Prevalence Survey was conducted in 2017–2018 to determine the actual burden of TB not only at the national level but also for three subnational strata (the states, regions other than Yangon, and the Yangon region) and develop a more efficacious country strategy on TB care and control. One hundred and thirty eight clusters were selected by population proportionate sampling. Adult (≥15 years of age) residents having lived for 2 weeks or more in the households of the selected clusters were invited to participate in the survey. The survey participants were screened for TB by a questionnaire and digital chest X-ray (CXR) after providing written informed consent. Individuals with a positive symptom screen and/or chest X-ray suggestive of TB were asked to provide sputum samples to test for *Mycobacterium tuberculosis* (Mtb) by Ziehl-Neelsen direct light microscopy, Xpert MTB/RIF Ultra (Xpert), and culture (Ogawa media). Bacteriologically confirmed TB cases were defined by an expert panel. Of 75 676 eligible residents, 66 480 (88%) participated, and 10 082 (15%) screened positive for TB. Among these, 322 participants were defined as bacteriologically confirmed TB cases. Cough lasting for two weeks or longer, one of the criteria used for screening for symptoms, could detect only 14% (45/322) of the study cases. The estimated prevalence of bacteriologically confirmed adult pulmonary TB was 468 (95% CI: 391–546) per 100,000. The prevalence was much higher among males, the older age group, urban Yangon and remote villages. In-depth interview with the participants on TB treatment showed that none of them was diagnosed in a TB health centre (primary care facilities). The prevalence of TB in Myanmar is still high due to challenges such as uncontrolled urbanization, an ageing population, migration, and poor access to health facilities in remote areas. New screening and diagnostic tools might help to detect more TB patients. There is a need to lay greater emphasis on multisectoral approaches, decentralization and the integration of basic TB services into primary care facilities.

## Introduction

Tuberculosis is one of the major public health problems globally. The World Health Organization (WHO) estimated that TB affected about 10 million people and claimed about 1.4 million lives in 2020 globally. Myanmar has been designated as one of the 30 countries with a high TB burden under the End TB Strategy of WHO, and bears a triple burden of drug-susceptible TB, drug-resistant TB and HIV-associated TB [1]. The estimated incidence of TB for 2017 by WHO before the survey was 358 (263–466)/100000 population, with 132,000 TB patients notified, which indicated that 25–30% of TB patients remain unnotified. In addition, the COVID-19 pandemic has reversed years of progress in the fight against TB with major challenges being providing and accessing essential tuberculosis services resulting in many people with TB were not diagnosed [1]. There was a 40-percent decrease in the number of detected TB cases in Myanmar in 2020 compared to last year [1]. Despite efforts to expand directly observed treatment (DOTS) in the 2000s, the National TB survey conducted in Myanmar in 2009‒2010 showed a very high TB burden with the bacteriologically confirmed TB prevalence of 613 (95% confidence interval: 502–748) per 100,000 population 15 years and above [2]. Since then, the National TB Program (NTP) and partners have been expanding TB service along with National TB Strategic Plan based on the Stop TB Strategy followed by the End TB Strategy [3]. Available funds for TB dramatically increased from USD 6–7 million a year in the late 2000s to USD 30–40 million or more a year in recent years. NTP has established quality-assured diagnostic centres in each township, the network of community TB care with the recruitment of community volunteers, and active case detection teams with portable chest X-rays (CXR) to reach the unreachable population in collaboration with partners. However, national TB surveillance data began to show a slow declining trend of case notification since 2013. While there were 148150 TB patients notified in 2012, it became 139625 in 2016 [4]. To determine the actual burden of TB and develop a more efficacious country strategy for TB care and control, the NTP conducted the fourth National TB Prevalence Survey. It also aimed to assess the change of TB burden, compared with the results of the 2009–2010 survey.

## Study population and methods

### Study design, sample size and sampling of clusters

Myanmar is divided into administrative subdivisions which include regions and states. Regions and states are divided into townships, which are further divided into wards in urban areas and village tracts in rural areas respectively (S1 Fig). We conducted a community-based, cross-sectional survey across all 17 states and regions covering people of the age of 15 years and above from October 2017 to September 2018 [5]. We designed the survey to estimate the prevalence not only at the national level but also in three sub-national areas namely the states (States), regions other than Yangon (Regions) and the Yangon region (Yangon).

Primary sample size for national estimate was calculated using following assumptions: (1) P = 429/100000 individuals aged 15 years or above, 30% reduction of bacteriologically confirmed TB since 2010, 613/100000; (2) k = 0.7 (0.69 in the 2009–2010 survey) corresponding design effect of 1.92; (3) Relative precision of 20%; (4) Cluster size = 500; and Participation rate = 85% (89% in the 2009–2010 survey). The estimated sample size was 54000 in 108 clusters (28 in States, 62 in Regions [other than Yangon] and 18 in Yangon). Since subnational estimates of TB prevalence seemed essential for developing a specific strategy addressing local epidemiological situations, Yangon and the states were allocated additional clusters to make the sample size 19 000 by 38 clusters, to have an independent prevalence estimate with a precision of less than 30%. Therefore, the final sample size was 69000 in 138 clusters.

Due to the differences in the administrative structure and the TB situation in urban and rural areas, each stratum was further divided into rural and urban clusters, in accordance with the proportion of the population. On the basis of the estimated population in the age group of 15 years and above, multistage probability proportional to size (PPS) was carried out in six substrata to select 138 clusters in total: states rural (28), states urban (10), regions rural (47), regions urban (15), Yangon rural (11) and Yangon urban (27) [S1 Fig]. Townships were selected first, followed by wards (52) in urban and village tracts (86) in rural.

We have also designated 70 “culture clusters” randomly in a proportion of population distribution to each sub-national stratum to conduct culture examination for every screening positive regardless of Xpert results to compare changes in the culture-positive prevalence between the 2009‒2010 survey and this 2017‒2018 survey. The surveys were similar in terms of screening criteria, sample collection, transportation to culture laboratories and culture examination, though we could not control factors such as improvement in infrastructure and road conditions that might have worked in favour of this survey. The number of culture *Mtb*-positive cases detected on the basis of a single specimen, using Ogawa media, was available in this survey, facilitating a direct comparison with the 2009–2010 survey. Therefore, we standardized the analysis of the culture results of morning specimens of the screening-positive participants of the two surveys.

### Sampling in a cluster and screening procedure

The sampling process was designed in accordance with WHO’s survey handbook [6]. People of the age of 15 years and above and who had been residing in the cluster for 14 days or longer were invited to participate in the study. All eligible people were invited for screening by individual interviews and CXR at the survey campsite set up temporally in the cluster area. In the interview, we asked the participants about their demographic characteristics, TB treatment history, TB symptoms, healthcare-seeking behaviours, including their utilization of public and private health facilities, and various TB risk factors, such as smoking and housing conditions by a structured interview. A direct digital CXR (posterior-anterior image) was taken, using a portable X-ray unit. If a woman was known or suspected to be pregnant, she was exempted from the CXR and sputum samples were collected if she had any symptoms suggestive of TB, regardless of the duration of the cough. The same applied to those who refused a CXR or were not able to get one taken, e.g. if they were bedridden. Every screening-positive participant was asked to submit three sputum samples: S1 or spot (immediately after the screening, at the survey site), S2 or morning (the next morning, at home) followed by S3 or morning spot. The survey participants were not tested for HIV as the inclusion of HIV testing in the community-based survey might have adversely affected voluntary participation. Only those diagnosed with TB were offered HIV testing in the health facility.

### Laboratory procedure

In all clusters, culture and smear tests were conducted only for those who had at least one positive result (including trace call) detected by Xpert Ultra due to constraints related to the capacity of the culture laboratories. Only in the 70 selected clusters (“culture clusters”) mycobacterial culture was conducted for every screened positive regardless of the Xpert Ultra results. The S1 and S3 specimens were sent to a designated township laboratory for Xpert MTB/RIF Ultra examination (Xpert Ultra) on the same day or the following day. The S2 specimens were sent to one of the pre-assigned culture laboratories in an icebox. The culture laboratory received specimens within 3 days after sample collection and carried out smear (LED fluorescent confirmed by ZN) and culture examination with Ogawa media without concentration when at least one Xpert result showed the presence of MTB, including trace call. Culture examination was done for all S2 specimens from 70 culture clusters.

### Survey case definitions

A participant was considered to have attended the survey if he or she was an eligible resident who went through the TB screening procedures. A participant was classified as “screened-positive” and thus eligible for sputum examination if the participant had at least one of the following: cough for two weeks or more or chest X-ray with abnormalities consistent with TB. Due to the possibly low positive predictive value of Xpert in the case of trace call and/or those with past TB with or without treatment, Xpert-based study cases definitions were revised to obtain the prevalence of TB. Therefore, the revised definition of a study TB case was screening-positive participants in whose case at least one of the two Xpert results showed the presence of Mtb, not including trace call (*Trace call was counted as “Negative*: *MTB not detected”)*, with at least one of the following conditions being met: 1) culture being Mtb-positive, and 2) deemed active TB without culture confirmation classified by a panel of experts from the Myanmar NTP based on chest X-ray images, and TB suggestive symptoms of each participant and treatment history. Xpert positive participants with TB treatment at the survey time were excluded from study cases and documented separately.

### Data management and analysis

The collection and management of data were both electronic and paper-based. Census data was collected and household data were entered into the survey database (Epi Info). The digital chest X-ray results in the individual survey forms and chest X-ray register (both paper-based) are entered into the survey database (Epi Info). Similarly, Xpert test results are stored in the Xpert database information using Electronic National Xpert Database information (GxAlert). The Xpert results were recorded in the individual survey forms and entered into the survey database (Epi Info). All smear, culture and identification results were recorded in the paper-based central laboratory register and entered into the survey database (Epi Info). The Lower Myanmar (LM) and Upper Myanmar (UM) data management units were responsible for collecting and integrating the data at the LM/UM level, and the central data management unit for the final integration and cleaning/validation of the data. The data were transformed into Stata version 9 (Stata Corp, Texas) for statistical analysis.

Frequency and percentage distributions, means, medians and range values were generated to define the survey data. The analysis took into account household data, data from individual interviews, data on healthcare-seeking behaviour, and CXR and laboratory data. Summary statistics were presented in tables and graphs generated with the help of Stata 14 and MS Excel. Three techniques were considered to estimate the prevalence of bacteriologically confirmed tuberculosis: (1) complete case analysis, which excludes participants with missing outcomes from both numerator and denominator; (2) inverse probability weighting (IPW), i.e., weight adjustment based on cluster allocation, cluster size, participation rate, etc.; and (3) multiple imputations (MI), in which missing values are imputed by considering the outcome and key variables. For the final estimates, the prevalence of Xpert-positive pulmonary TB among adults of the age of 15 years and above was calculated according to the WHO guidelines, with a complete case analysis with IPW (Model 1) and MI+IPW (Model 3) with further adjustment for population structure of population projection from pop census [7]. The association of predictive factors with the prevalence of TB was determined using logistic regression analysis. Inverse probability weights were applied to the survey participants and the analysis accounted for the clustering of individuals. For the multivariable analysis to calculate the adjusted odds ratio, the random effect logit model with all variables (Model 2) and the random effect logit model with variables with a p-value of more than 0.05 in crude analysis (Model 1) were used.

### Quality management

While the survey steering committee oversaw the progress of the study, members of the technical advisory committee regularly visited the cluster operation with NTP central and WHO national staff. The data entry e-form was designed with an auto-check system of data consistency; all chest X-ray images were reviewed by central X-ray readers within a week to give filed readers feedback; all Xpert units were wired and results were transmitted automatically. In collaboration with US-CDC (Centers for Disease Control and Prevention), Research Institute of Tuberculosis, Japan Anti-Tuberculosis Association (RIT/JATA) and Global Task Force on TB Impact Measurement, the WHO Myanmar office organized an international monitoring mission in the early and mid-stages of the survey and conducted data review monitoring after completing the survey.

### Ethical statement

This study was given scientific and ethical approval by the Ethics Review Committee, Department of Medical Research, Ministry of Health and Sports, Myanmar (115/2017). Members of WHO’s Global Task Force on TB Impact Measurement from US-CDC, RIT/JATA and WHO reviewed the protocol. All invited participants were informed about the risks and benefits of the study. Only those who submitted written consent were counted as study participants. The local TB program provided individual patient management in consultation with technical committee members, when required, independently from the final study results. Although we did not invite children to the study, children living in the same house with TB patients detected by the survey were given contact assessments including chest X-rays when required.

## Results

### Participations

**Fig 1:**
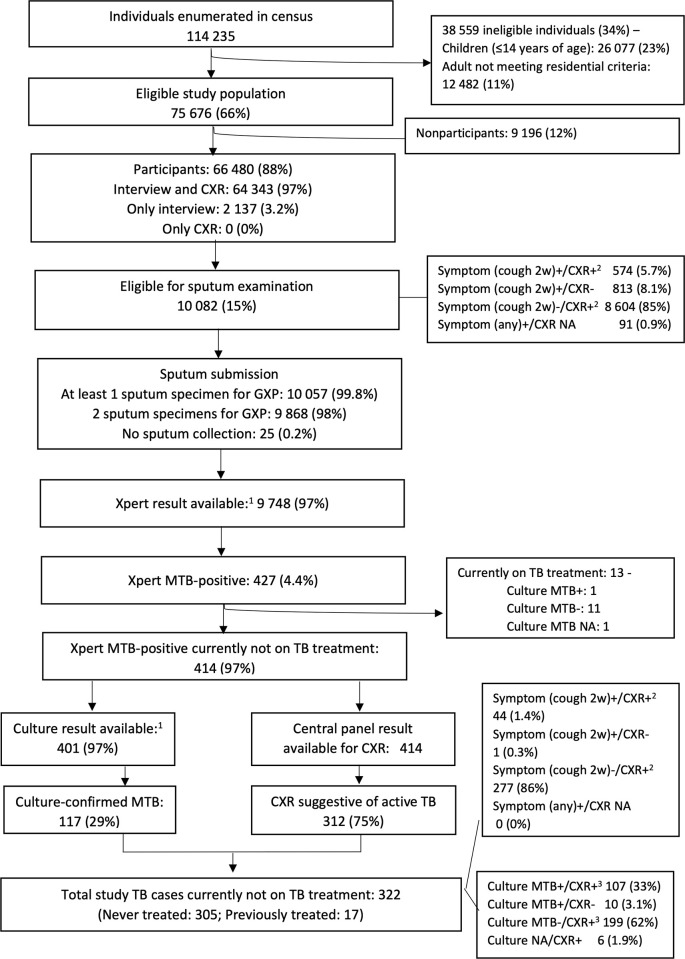
Flow diagram of the 4^th^ TB Prevalence survey in Myanmar, 2017–2018.

**Fig 2:**
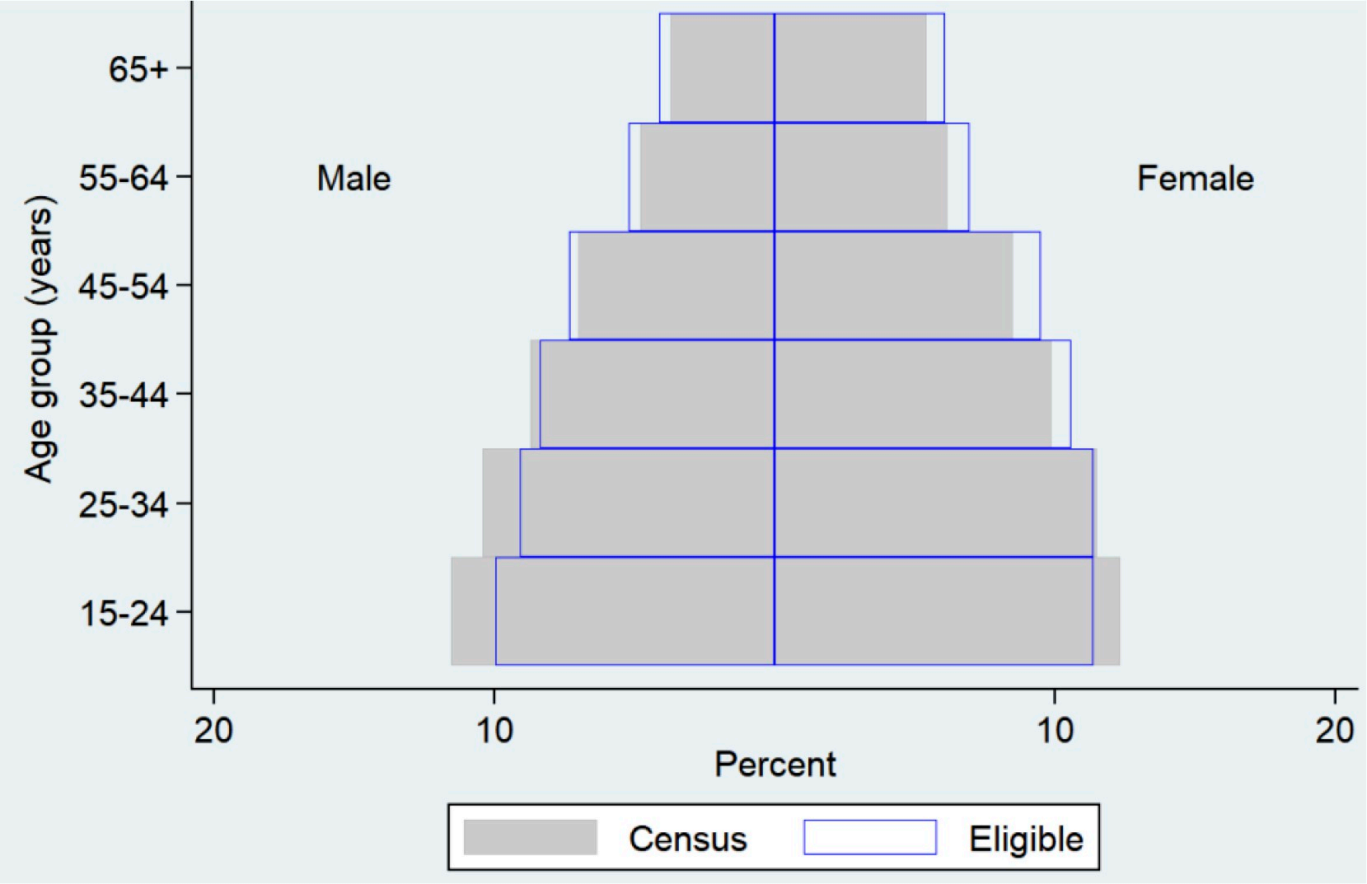
Comparison of age-sex distribution for the census and eligible population.

**Fig 3:**
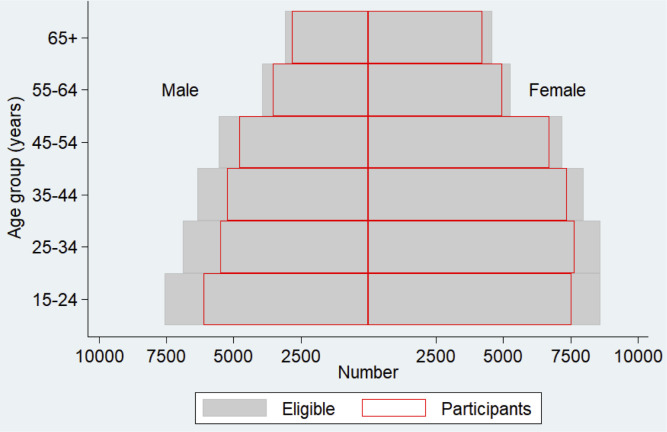
Comparison of age-sex distribution for the eligible and participated population.

In the 138 clusters, 24 320 households were visited and assessed. A total of 114 235 people were enumerated and documented, regardless of their age and physical presence on the day of the census (Figs 1 and 2). Children under the age of 15 years comprised 23% (26 077) of the enumerated people; In all, 75 676 people (66% of enumerated) were eligible for the survey (Figs 1 and 2). Young age groups (females of 15‒24 years of age and males of 15‒34 years of age) were less likely to be eligible (Fig 2). Of the 75 676 persons eligible for the study, 66 480 (88%) participated in the study (Figs 1 and 3). The participation rate was higher among females (91%) than males (84%), and older age groups than young age groups (Fig 3). The average number of participants was 482 per cluster. The overall participation rate of 88% was almost the same as that in the 2009‒2010 survey (89%) [S1 Table].

### Screening and laboratory results

Of the total of 66 480 participants, 96 (0.1%) were on TB treatment at the time of the survey and 2961 (4.5%) had a history of previous treatment for TB. Of the 66 480 participants, 1387 (2.1%) reported having a cough for more than two weeks (Table 1). A total of 10 082 (15%) were screening-positive and eligible for sputum examination (Fig 1). Of these, 574 were both symptom-positive and CXR-positive; 813 were only symptom-positive; 8604 were only CXR-positive and 91 had a TB-related symptom, but were exempted from undergoing a CXR (Fig 1). Of the 10 082 participants eligible for sputum examination, 10 057 (99.8%) submitted at least one sputum specimen and 9868 (98%) submitted at least two specimens (Fig 1). The sputum collection rate was very high in this survey. The Xpert results were available for 9748 (97%). In all 138 clusters, of the 427 participants with at least one specimen showing Xpert MTB-positive (excluding trace), 117 (27%) were confirmed to be Mtb-positive by culture (Fig 1, Table 2).

**Table 1:** Sociodemographic characteristics of study participants.

		National		State		Region		Yangon	
		Number	%	Number	%	Number	%	Number	%
Total participants		66 480		18 119		30 532		17 829	
Education	Illiterate	4 744	7.1	2 784	15	1 451	4.8	509	2.9
	Can read/write	4 620	6.9	1 074	5.9	2 869	9.4	677	3.8
	Primary school	22 415	34	5 770	32	12 568	41	4 077	23
	Middle school	14 770	22	3 711	20	6 528	21	4 531	25
	High school	12 502	19	3 235	18	4 709	15	4 558	26
	College and above	2 436	3.7	1 535	8.5	2 362	7.7	3 476	19
	Other	4 937	7.4	10	0.1	45	0.1	1	0.0
Ethnicity	Kachin	1 479	2.2	1 421	7.8	24	0.1	34	0.2
	Kayah	573	0.9	559	3.1	4	0.0	10	0.1
	Kayin	4 615	6.9	2 264	12	1 864	6.1	487	2.7
	Chin	604	0.9	478	2.6	27	0.1	99	0.6
	Mon	1 449	2.2	1 254	6.9	36	0.1	159	0.9
	Bamar	45 641	69	3 521	19	27 628	90	14 492	81
	Rakhine	3 405	5.1	2 908	16	38	0.1	459	2.6
	Shan	5 950	9.0	5 106	28	709	2.3	135	0.8
	Other	2 764	4.2	608	3.4	202	0.7	1 954	11
Religion	Buddhist	61 871	93	15 455	85	30 079	99	16 337	92
	Christian	2 791	4.2	2 211	12	213	0.7	367	2.1
	Hindu	116	0.2	20	0.1	4	0.0	92	0.5
	Muslim	1 693	2.5	430	2.4	233	0.8	1 030	5.8
	Other	9	0.0	3	0.0	3	0.0	3	0.0
Marital Status	Single	17 532	26	4 238	23	7 672	25	5 622	32
	Married	42 727	64	12 075	67	20 073	66	10 579	59
	Separated/divorced	1 064	1.6	372	2.1	421	1.4	271	1.5
	Widowed	5 146	7.7	1 428	7.9	2 365	7.7	1 353	7.6
	No answer	11	0.0	6	0.0	1	0.0	4	0.0
Occupation	Professional and technical worker	2 398	3.6	529	2.9	993	3.3	876	4.9
	Owner of business	5 037	7.6	1 237	6.8	2 335	7.6	1 465	8.2
	Merchant	1 956	2.9	595	3.3	704	2.3	657	3.7
	Service provider (including government servant)	3 415	5.1	484	2.7	664	2.2	2 267	13
	Sales person	1 417	2.1	430	2.4	345	1.1	642	3.6
	Engaged in agriculture, animal husbandry, forestry, fishing, hunting	14 384	22	5 448	30	8 027	26	909	5.1
	Production-related or transport worker, equipment operator, labourer	1 951	2.9	290	1.6	622	2.0	1 039	5.8
	Other worker	8 628	13	2 125	12	4 941	16	1 562	8.8
	Housewife	9 113	14	2 657	15	4 627	15	1 829	10
	Student	3 998	6.0	1 092	6.0	1 575	5.2	1 331	7.5
	Dependant	12 588	19	2 880	16	5 123	17	4 585	26
	Religious occupation	149	0.2	65	0.4	26	0.1	58	0.3
	Other	1 446	2.2	287	1.6	550	1.8	609	3.23.24
Age group	15‒24	13654	20.5	3 862	21.3	5 654	18.5	4 138	32
	25‒34	13151	19.8	3 453	19.1	6 024	19.7	3 674	20.6
	35–44	12609	20	3 330	18.4	6 014	19.7	3 265	18.3
	45–54	11513	17.3	3 259	18	5 463	17.9	2 791	15.6
	55–64	8484	12.8	2 383	13.2	3 957	13	2 144	12
	≥ 65	7069	10.6	1832	10.1	3 420	11.2	1 817	10.2
Sex	Female	38 495	57.9	10 475	57.8	17 556	57.5	10 464	58.7
	Male	27 985	42.1	7 644	42.2	12 976	42.5	7 365	41.3

**Table 2:** Study TB cases after Xpert MTB, culture and clinical panel decision.

	Number
**At least 1 Xpert MTB/Rif+ (excluding trace)**	**427**
Eligible for culture examination	427
Currently not on TB treatment	414
Currently not on TB treatment, with culture results	405
MTB-positive	117
NTM	2
MTB-negative	282
Contamination	4
Deemed active TB by clinical panel decision	312
**Xpert MTB-positive confirmed positive by culture**	**117**
**Deemed active TB by clinical panel decision without culture confirmation**	**205**
**Xpert-positive active pulmonary TB (not on treatment)**	**322**

MTB–*Mycobacterium tuberculosis*, NTM–non tuberculosis mycobacteria.

### Study TB cases

Of the 405 participants whose culture results were available and who were not currently on TB treatment, Mtb isolates were detected by culture examination in the case of 117 (Table 2). As for those whose culture result was not confirmed, the central clinical panel defined 205 as active TB cases (Fig 1, Table 2). They had at least one Xpert MTB-positive result and a CXR suggestive of active TB disease. In all, 322 were categorized as Xpert-positive active pulmonary TB cases (Table 2).

Of the 322 study cases, eighty-six (27%) of the cases were female and 236 (73%) male (Table 3). Nationwide, 45% of the study cases were from the older age group (55 years or older (Table 3). There were more study cases from this age group, especially in the states and regions other than Yangon. Cough lasting for two weeks or longer, one of the criteria used for screening for symptoms, could detect only 14% (45/322) of the study cases (Table 3). Even when the screening criteria were expanded to the presence of any TB symptom, only half of the prevalent cases could be detected. As for screening by CXR, both field and central readings were able to detect almost all study cases (field: 99.7%, central: 97%). The majority of the study cases (95%) had no history of TB treatment (Table 3). The cases included 10 (3.1%) who were rifampicin resistant, 7 new cases and 3 who had been treated previously (Table 3).

**Table 3:** Characteristics of study TB cases.

		Total		State		Region		Yangon	
		Number	%	Number	%	Number	%	Number	%
	Study case	322		65		149		108	
Sex	Male	236	73	46	71	112	75	78	72
	Female	86	27	19	29	37	25	30	28
Age group	15‒24	24	7.5	5	7.7	6	4.0	13	12
	25‒34	37	12	5	7.7	17	11	15	14
	35‒44	59	18	11	17	26	17	22	20
	45‒54	58	18	12	19	26	17	20	19
	55‒64	69	21	14	22	38	26	17	16
	65‒74	47	15	13	20	18	12	16	15
	75+	28	8.7	5	7.7	18	12	5	4.6
Symptom	Cough ≥ 2 weeks	45	14	13	20	20	13	12	11
	Any TB-related symptom(s)	153	48	34	52	69	46	50	46
CXR	Field screening-positive	321	99.7	64	99	149	100	108	100
	Central screening-positive	313	97	63	97	146	98	104	96
	Active TB by central reading	267	83	57	88	118	79	92	85
TB history	No TB treatment history	305	95	62	95	145	97	98	91
	Previously treated	17	5.3	3	4.6	4	2.7	10	9.3
Laboratory result	Smear-positive	42	13	14	22	14	9.4	14	13
	Culture *M*.*tb*-positive	117	36	34	52	42	28	41	38
	Rifampicin-resistant	10	3.1	3	4.6	3	2.0	4	3.7
	*New case*	*7*	*2*.*2*	*2*	*3*.*1*	*2*	*1*.*3*	*3*	*2*.*8*
	*Previously treated*	*3*	*0*.*9*	*1*	*1*.*5*	*1*	*0*.*7*	*1*	*0*.*9*

CXR–chest X-ray, Mtb–*Mycobacterium tuberculosis*.

### Prevalence of Xpert MTB-positive pulmonary TB

**Fig 4:**
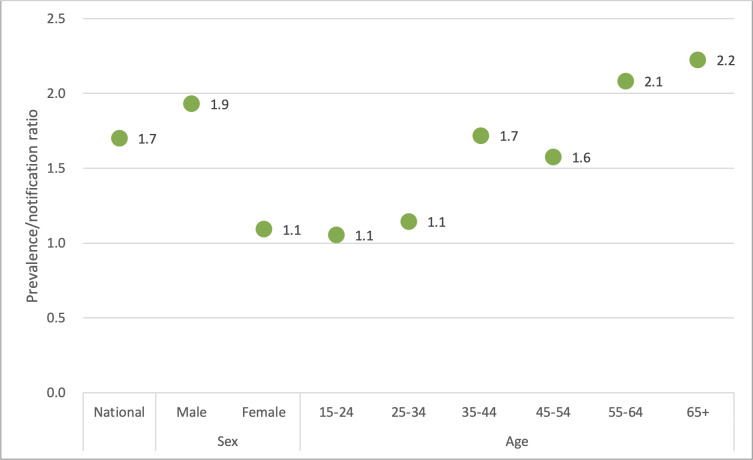
Prevalence-to-case notification (P/N) ratios, Myanmar.

The prevalence of Xpert MTB-positive pulmonary TB among adults of the age of 15 years and above was 468 per 100 000 population after adjusting for the estimated population structure in 2018 (S2 and S3 Tables). The case notification rate in 2018 was 272 (105 913/38 976 201 population*100 000) that includes new and relapse, pulmonary DS and DR-TB cases in adults (≥15 years old). Comparing prevalence (identified in the survey) with notifications (identified through National TB Programme) as a prevalence-to-notification (P/N) ratio provides a measurable indicator of the effectiveness of case detection. The higher the P/N ratio, the longer the time taken for a prevalent case to be notified to the NTP. The national P/N ratio was 1.7 (Fig 4). It was much higher for males (1.9) than females (1.1), and for the older age groups than the younger age groups (Fig 4 and S4 Table).

### Change of prevalence of culture positive TB since 2010

The prevalence of cases found culture Mtb-positive using one specimen was 256 (95% CI: 173‒339) per 100 000 population. When we standardized the results of the 2009‒2010 survey, the reported prevalence (602/100 000), using two culture specimens, became 520 (95% CI: 415‒624) per 100 000 population. The decrease from 520 to 256 represented a 51% reduction in the prevalence of culture-confirmed pulmonary TB.

### Predictive factors to be prevalent TB patients

To determine the association of various factors with the Xpert-positive cases (322), the crude odds ratio and adjusted odds ratio were calculated (Table 4). The factors were administrative area (states, regions other than Yangon, Yangon), type of area (urban, rural), sex, age group, religion, ethnicity, education status, occupation, history of TB treatment, contact history of TB patient, smoking status, and alcohol status (Table 4). A random-effects logit regression model was used to evaluate the association of these factors. Based on the adjusted odds ratios, the factors associated with testing Xpert-positive were male sex, age of 35 years and above, history of contact with TB patients and current smokers (Table 4).

**Table 4:** Analysis of predictive factors: Geographical and sociodemographic characteristics.

		Xpert-positive study case			Crude odds ratio				Model 1: Random effect logit model with all variables (adjusted odds ratio)				Model 2: Random effect logit model with variables with p<0.05 in crude analysis (adjusted odds ratio)			
		Cases	Participants	Rate per 100 000 population	Odds ratio	95% CI LL	95% CI UL	p-value	Odds ratio	95% CI LL	95% CI UL	p-value	Odds ratio	95% CI LL	95% CI UL	p-value
Stratum	Region	149	30 407	490.0	1.36	1.02	1.82	0.038	1.12	0.71	1.75	0.633	1.09	0.70	1.71	0.703
	State	65	18 045	360.2	Reference				Reference				Reference			
	Yangon	108	17 694	610.4	1.70	1.25	2.31	0.001	1.46	0.89	2.37	0.130	1.41	0.87	2.28	0.161
Rural/urban	Rural	186	42 157	441.2	Reference				Reference				Reference			
	Urban	136	23 989	566.9	1.29	1.03	1.61	0.026	1.23	0.87	1.73	0.244	1.19	0.85	1.65	0.307
Sex	Female	86	38 369	224.1	Reference				Reference				Reference			
	Male	236	27 777	849.6	3.81	2.98	4.89	0.000	2.59	1.89	3.56	0.000	2.53	1.86	3.45	0.000
Age group	15‒24	24	13 641	175.9	Reference				Reference				Reference			
	25‒34	37	13 109	282.2	1.61	0.96	2.69	0.071	1.49	0.88	2.50	0.135	1.49	0.89	2.51	0.131
	35‒44	59	12 536	470.6	2.68	1.67	4.31	0.000	2.49	1.54	4.03	0.000	2.51	1.55	4.05	0.000
	45‒54	58	11 421	507.8	2.90	1.80	4.66	0.000	2.68	1.65	4.35	0.000	2.70	1.67	4.38	0.000
	55‒64	69	8 424	819.1	4.69	2.94	7.46	0.000	4.29	2.66	6.92	0.000	4.34	2.70	6.98	0.000
	65+	75	7 015	1 069.1	6.13	3.87	9.72	0.000	5.94	3.67	9.61	0.000	6.07	3.77	9.78	0.000
Religion	Non-Buddhist	14	4 582	305.5	Reference				Reference							
	Buddhist	308	61 564	500.3	1.64	0.96	2.81	0.071	1.28	0.69	2.36	0.439				
Ethnicity	Non-Burmese	75	20 732	361.8	Reference				Reference				Reference			
	Burmese	247	45 414	543.9	1.51	1.16	1.95	0.002	1.32	0.90	1.94	0.160	1.36	0.95	1.97	0.096
Education	Literate	297	61 439	483.4	Reference				Reference							
	Illiterate	25	4 707	531.1	1.10	0.73	1.66	0.650	1.23	0.79	1.93	0.362				
Occupation^1^	Non-farmer	245	51 816	472.8	Reference				Reference							
	Farmer	77	14 330	537.3	1.14	0.88	1.47	0.326	1.02	0.75	1.39	0.907				
Previous treatment	No	305	63,240	482.3	Reference				Reference							
	Yes	17	2 906	585.0	1.21	0.74	1.98	0.437	0.86	0.52	1.42	0.556				
Contact^2^	No	296	63 214	468.3	Reference				Reference				Reference			
	Yes	26	2 932	886.8	1.90	1.27	2.84	0.002	1.83	1.21	2.76	0.004	1.83	1.21	2.76	0.004
Smoking	Never smoked	125	48 368	258.4	Reference				Reference				Reference			
	Ex-smoker	35	3 596	973.3	3.79	2.60	5.53	0.000	1.49	0.98	2.25	0.060	1.48	0.98	2.23	0.064
	Current smoker	162	14 182	1 142.3	4.46	3.53	5.64	0.000	2.42	1.84	3.18	0.000	2.44	1.86	3.21	0.000
Alcohol	Never drank	179	51 608	346.8	Reference				Reference				Reference			
	Ex-drinker	47	3 500	1 342.9	3.91	2.83	5.40	0.000	1.15	0.80	1.66	0.456	1.14	0.79	1.65	0.476
	Current drinker	96	11 038	869.7	2.52	1.97	3.23	0.000	1.13	0.84	1.54	0.415	1.14	0.84	1.54	0.408
Symptom	No symptom	169	49 455	341.7	Reference								Reference			
	Any TB symptom	108	15 057	717.3	2.10	1.65	2.67	0.000					1.63	1.25	2.13	0.000
	Cough ≥2 weeks	45	1 314	3 424.7	10.02	7.18	13.99	0.000					3.04	2.07	4.47	0.000
Central CXR	Normal/abnormal, not eligible for sputum examination	9	58 143	15.5	Reference								Reference			
	Active/possible TB	306	2 757	11 099.0	717.03	369.14	1 392.81	0.000					564.60	289.76	1100.13	0.000
	Healed TB/other abnormality	7	2 697	259.5	16.77	6.24	45.06	0.000					12.05	4.46	32.56	0.000
BMI	<15.0	25	1 009	2 477.7	5.72	3.74	8.76	0.000					2.02	1.24	3.28	0.005
	15.0‒18.4	117	10 171	1 150.3	2.66	2.09	3.37	0.000					1.61	1.24	2.09	0.000
	18.5‒24.9	163	37 659	432.8	Reference								Reference			
	25.0‒29.9	15	12 658	118.5	0.27	0.16	0.46	0.000					0.39	0.22	0.67	0.001
	30.0+	2	4 278	46.8	0.11	0.03	0.44	0.002					0.18	0.04	0.75	0.018

^1^ Farmer includes those engaged in agriculture, animal husbandry and forestry, fishing and hunting.

^2^ History of contact with TB patient in the last 2 years.

Abbreviations: UL–Upper limit; LL–Lower limit.

### Health seeking behaviours

Of the 96 participants on treatment, 67 (70%) were diagnosed by public health facilities (township health/TB center, station/township hospital, public hospital) and 87 (91%) were on medication provided by them (township health/TB center or rural/urban health center) (Table 5). Of the 1387 participants who reported having a cough for more than two weeks, 748 (54%) had sought care for their symptoms (S5 Table). In all three strata, most symptomatic participants visited the clinic of a private practitioner for their first consultation: 43% in Yangon, 31% in regions other than Yangon and 29% in the states (S5 Table). While the second most popular site for seeking healthcare was pharmacies in Yangon (34%) and regions other than Yangon (28%), it was health centres in the states (27%). The use of the public health sector was rather common in the states (43%) and regions other than Yangon (33%), but not so much in Yangon (15%) (S5 Table). The survey team asked the 639 participants who had not sought care for their symptoms to explain their behaviour (S6 Table). Nationwide, the most common answers were “self-treatment” (44%) and “ignored” (41%). The majority of these were from states and regions other than Yangon (S6 Table).

**Table 5:** Type of facility used by participants currently on TB treatment.

	National		State		Region		Yangon	
	Number	%	Number	%	Number	%	Number	%
Participants currently on TB treatment	96		23		31		42	
Where were you diagnosed with TB?								
Township health/TB center, station/township hospital	55	57	16	70	17	55	22	52
Public hospital (district/region/state)	12	13	2	8.7	5	16	5	12
Rural/urban health center	0	0.0	0	0.0	0	0.0	0	0.0
Private practitioner’s clinic	15	16	3	13	4	13	8	19
Private hospital	8	8.3	2	8.7	2	6.5	4	9.5
INGO clinic	4	4.2	0	0.0	2	6.5	2	4.8
Mobile clinic	1	1.0	0	0.0	0	0.0	1	2.4
Other	1	1.0	0	0.0	1	3.2	0	0.0
Where are you taking the treatment?								
Township health/TB center	59	62	17	74	22	71	20	48
Public hospital	9	9.4	2	8.7	1	3.2	6	14
Rural/urban health center	19	20	2	8.7	5	16	12	29
Private practitioner’s clinic	3	3.1	2	8.7	0	0.0	1	2.4
Private hospital	2	2.1	0	0.0	0	0.0	2	4.8
Other	4	4.2	0	0.0	3	9.7	1	2.4

INGO–International non-government organisations.

## Discussion

The fourth National TB Prevalence Survey in Myanmar had a unique design. Estimates of prevalence were sought not only for the national level, but also for three subnational strata: the states, regions other than Yangon, and the Yangon region. The survey used new diagnostic technology, GeneXpert, whereas the previous survey relied on the diagnostic tools of smear microscopy and solid culture. However, it employed the same screening and diagnostic methods ‒ screening by symptoms and CXR and solid culture as the 2009‒2010 survey, to facilitate a direct comparison between the results of the two surveys. The survey design, according to which those who were TB presumptive by symptom and/or CXR were asked to undergo two Xpert Ultra tests, had programmatic implications for the NTP, which could consider replacing smear microscopy with a rapid molecular test. Assuming that the sensitivity of the Xpert Ultra test is higher than that of solid culture, the study aimed to obtain national and subnational estimates for the prevalence of Xpert-positive-TB. The prevalence of culture-positive TB could be compared with that of the 2009‒2010 National TB Prevalence Survey with methodological adjustments.

The survey showed that the prevalence of TB is still high in Myanmar, though it suggested that there has been a significant reduction since 2009‒2010. According to the results, the current TB burden at the national level is close to the burden estimated by WHO [1, 4]. Further, the subnational surveys shed light on the different characteristics of the TB epidemic, particularly in Yangon.

322 participants were identified as Xpert MTB-positive cases who had not received treatment at the time of the survey. In the community of the 138 clusters, 96 TB patients were receiving treatment on the day of the survey. These 96 were diagnosed mostly by routine passive case detection by symptom. The fact that the number of unidentified TB patients in the community was greater than the number on treatment suggests that there are delays in diagnosis and that many people remain in a bacteriologically positive condition without a TB diagnosis for a long duration of time. Screening by symptoms had a very limited role in case detection as only 20% of the study TB cases in the states, 13% in regions other than Yangon, and 11% in Yangon reported chronic cough lasting two weeks or longer during the survey interview. It is possible that the existing TB services have been identifying TB patients with a chronic cough efficiently [12]. The central CXR reading diagnosed 83% of the study cases as cases of active TB disease, thus, CXR plays an essential role in screening community members for TB as identified in the prevalence survey in Vietnam [8]. Effective case detection may facilitate and hasten a decline in the incidence of TB by reducing transmission [9]. The low prevalence of symptomatic bacteriologically positive TB suggests that very few symptomatic TB patients who would be smear-positive and could be identified by the classical diagnostic method remain in most places or among most populations excluding inaccessible/hard-to-reach populations, such as the elderly, and populations in remote villages and urban slums with migrants.

The National TB Prevalence Survey showed that the prevalence of Xpert-positive pulmonary TB in Myanmar was still high: 468 (391–546) per 100 000 population among adults of the age of 15 years and above. This prevalence is higher compared to several other surveys done in high TB-burden countries in the region over the last 5 years, for example, Bangladesh (287/100 000 adults, CI: 244–340) in 2015 [10], Nepal (375/100 000 adults, CI: 307–441) in 2018 [11], and Vietnam (322/100 000 adults, CI: 250–399) in 2017 [8]. Men were more likely to get TB and less likely to be diagnosed and treated. This can also be seen by the high P/N ratio in males and the elderly population, suggesting a delay in the diagnosis of TB is more serious among men and the older age groups. The high P/N ratio in males was also identified in the surveys done in Pakistan [12] and the Philippines [13].

The study showed that smoking was the biggest preventable risk factor for TB. The adjusted odds ratio for smoking was even higher than that for TB contacts. Interventions to stop smoking not only among patients but also in the community can become effective tools for reducing the prevalence of TB as the issue of smoking, which is traditionally highly prevalent among men, is now increasing among women. The prevalence increased with age. Among the oldest age group (65 years or older), it exceeded 1% and was seven times higher than that among the youngest age group. More than half of the study TB cases (172/322) were in the age group of 50 years and above. The greater proportion of older TB patients might indicate an epidemiological shift and imply that there are more cases of reactivation of disease or remote infection than cases of new infection. It might also suggest that older patients have poor access to TB diagnostic services.

The prevalence of TB in the Yangon region is significantly higher than those in the states and other regions, despite better access to health services. The survey found that the transmission rate seemed to be high in congested urban areas, consistent with previous findings [14]. As Yangon is the economic and transportation hub of Myanmar, the movement of the population from the rural and remote areas is resulting in rapid changes in its environment [15]. The survey showed that for their first consultation to seek care, 79% of the symptomatic participants in Yangon chose the private sector (private practitioner’s clinic, private hospital or pharmacy). This calls for the strengthening of PPM activities, including the approach to drug sellers and pharmacies such as improving pharmacy practice about TB control including inadequate questioning of symptomatic patients and lack of referral for testing through the public-private mix (PPM) initiatives to engage pharmacies and drug sellers into national TB programmes to improve case detection [16, 17]. Urban-specific strategies for TB control should be established to support these patients.

The study also showed that none of the participants on treatment was diagnosed in a TB health centre, and relatively few received TB treatment from these centres in the states and regions other than Yangon. This means that basic TB services need to be expanded further and integrated into primary care facilities, including station hospitals and health centres, in the rural and remote areas [18, 19]. Although primary health-care centres are often major providers of TB services in other Asian countries, the interviews of the survey participants clearly indicated that primary care services have little to offer by way of TB case detection and treatment in Myanmar. Under the DOTS strategy, midwives played a major role in providing support to TB patients. However, the restructuring of public health services ushered in changes in the tasks of basic health workers. It is necessary to provide clear guidance on the roles of primary-level health-care facilities and basic health workers and to define the link between community volunteers and primary care facilities. It is essential to introduce services for TB screening and treatment in primary health-care facilities, just as the noncommunicable disease programme has introduced care for the elderly in these facilities. Whereas the younger generation can travel to the townships to seek care, elderly patients may remain in the community without seeking care for a long time, since it is hard for them to travel. Basic TB services should be decentralized to the primary level and integrated into primary health-care facilities.

### Limitations of the survey and analysis

Although we conducted the survey successfully, the following issues should be noted as limitations of the study: (1) Ten of 138 clusters had to be replaced mostly due to security concerns in rural areas in States. To prevent a selection bias due to convenient sampling, the alternative sites within the same township were selected by PPS. The existence of areas affected by conflict poses a challenge to TB care and prevention in Myanmar. The prevalence of TB may be higher in these sites due to poor access to TB services. On the other hand, the low population density in these areas may prevent the spread of TB and make for a lower incidence.; (2) Young males had a lower participation rate of 80% and this might have been due to the duration of screening days, as it always took place on weekdays, when eligible residents had to work or study, which may have impeded the participation in younger age groups. To minimise the bias caused by this, the survey teams worked extra time to perform screening procedures in the evening and encouraged younger eligible residents to participate in the survey after their daily work and study; (3) Capacity limitation of culture laboratories due to rapid expansion of MDR-TB service restricted the study design and the survey could not offer culture examination to every screening-positive participant; (4) There was no clear international consensus to deal Trace Call result by Xpert Ultra in TB prevalence survey where false positive among those with previous TB history concerns. We dropped them from “MTB detected” in this study; (5) We didn’t aim to obtain the prevalence of smear-positive TB due to the limitation of the sample size. We used smear examination as one of the quality assessment tools in the laboratory and may have impacted the specificity and sensitivity of the diagnosis; and (6) As the nature of the TB prevalence survey, there was no assessment on extrapulmonary TB or childhood TB, suggesting that extrapolation of estimates to all ages and all forms of TB relied on child TB surveillance or research data in Myanmar.

## Conclusions

The National TB Prevalence Survey 2017–2018 indicated that the efforts to reduce the TB burden in Myanmar have had a significant impact. The lessons learnt from the findings of the 2009‒2010 survey were utilized effectively to improve TB care and control. The increased investment was made in collaboration with international financial and technical partners. However, the 2017‒2018 survey also found that the burden of TB in Myanmar is still high. In fact, the observed prevalence by new technologies is even higher than that was expected, and the problem is compounded by emerging challenges such as unplanned urbanization, migration and an ageing population coupled with the current COVID-19 pandemic. It is essential to adopt wider or multisectoral approaches. The NTP needs to prioritise the incorporation of new screening and diagnostic tools with higher sensitivity to detect more TB patients and further decentralization of basic TB services and their integration into primary care facilities. This would facilitate the care of the high-risk groups including the elderly.

## Supporting information

S1 Fig Distribution of the survey clusters across Myanmar (N = 138 clusters). Orange 1 cluster, Blue 2 clusters and Green 3 clusters and Myanmar administrative Structure.
(TIFF)

S1 Table Study participants. (DOCX)

S2 Table Comparison between Xpert and culture results in 70 clusters. (DOCX)

S3 Table National prevalence rate of Xpert-positive pulmonary TB (≥ 15 years old). (DOCX)

S4 Table Prevalence-to-case notification (P/N) ratios, 2017–2018 NTPS, Myanmar. (DOCX)

S5 Table Preferred facility for first consultation among symptomatic participants. (DOCX)

S6 Table Reasons for not seeking health care. (DOCX)

## References

[1] Global Tuberculosis Report 2021. Geneva: WHO; 2021. https://apps.who.int/iris/rest/bitstreams/1379788/retrieve

[2] Report on National TB Prevalence Survey 2009–2010, Myanmar. Ministry of Health, Government of Myanmar (https://www.who.int/tb/advisory_bodies/impact_measurement_taskforce/prevalencesurveymyanmar_2009-10report.pdf

[3] National Tuberculosis Programme, Myanmar. National Strategic Plan for Tuberculosis 2016–2020. Ministry of Health and Sports, Government of Myanmar https://themimu.info/sites/themimu.info/files/assessment_file_attachments/National_Strategic_Plan_for_Tuberculosis_2016-2020.pdf

[4] Annual Report 2016, National Tuberculosis Programme, Myanmar; June 2018 https://mohs.gov.mm/Main/content/publication/tuberculosis-annual-report-2016-by-national-tuberculosis-programme-myanmar

[5] Myanmar Information Management Unit. The 2014 Myanmar Population and Housing Census https://themimu.info/census-data

[6] Tuberculosis prevalence surveys: a handbook, 2007. Geneva: WHO; 2007. https://www.who.int/tb/advisory_bodies/impact_measurement_taskforce/resources_documents/thelimebook/en/

[7] Seaman SR, White IR, Copas AJ, Li L: Combining multiple imputation and inverse-probability weighting. Biometrics. 2012, 68 (1): 129–137. doi: 10.1111/j.1541-0420.2011.01666.x 22050039 PMC3412287

[8] Nguyen HV, Tiemersma EW, Nguyen HB, Cobelens FGJ, Finlay A, Glaziou P, et al. The second national tuberculosis prevalence survey in Vietnam. PLOS One. 2020 15(4):e0232142 doi: 10.1371/journal.pone.0232142 32324806 PMC7179905

[9] Khaing PS, Kyaw NTT, Satyanarayana S, Oo NL, Aung TH, Oo HM, et al. Treatment outcome of tuberculosis patients detected using accelerated vs. passive case finding in Myanmar Int J Tuberc Lung Dis. 2018 22(10):1145–1151. doi: 10.5588/ijtld.18.0038 30236181

[10] Directorate General of Health Services, Ministry of Health and Family Welfare (Bangladesh), National Tuberculosis Control Program (NTP) (Bangladesh), Bangladesh National Tuberculosis Prevalence Survey 2015–2016. Dhaka, Bangladesh. https://drive.google.com/file/d/1zNDPqQDhvLDv_tbjq0_bvthJktNd4Uns/view

[11] Survey protocol, National Tuberculosis Prevalence Survey 2018/2019, Nepal. National Tuberculosis Centre, Department of Health Services, Ministry of Health and Population, Thimi, Bhaktapur, Nepal. https://nepalntp.gov.np/wp-content/uploads/2020/03/TBPS-Factsheet-English.pdf

[12] Qadeer E, Fatima R, Yaqoob A, Tahseen S, Ul Haq M, Ghafoor A, et al. Population Based National Tuberculosis Prevalence Survey among Adults (>15 Years) in Pakistan, 2010–2011. PLoS One. 2016 11(2):e0148293. doi: 10.1371/journal.pone.0148293 26863617 PMC4749340

[13] Lansang MAD, Alejandria MM, Law I, Juban NR, Amarillo MLE, Sison OT, et al. High TB burden and low notification rates in the Philippines: The 2016 national TB prevalence survey. PLoS One. 2021 16(6):e0252240. doi: 10.1371/journal.pone.0252240 34086746 PMC8177416

[14] Aung HL, Nyunt WW, Fong Y, Biggs PJ, Winkworth RC, Lockhart PJ, et al. Genomic Profiling of Mycobacterium tuberculosis Strains, Myanmar. Emerg Infect Dis. 2021 27(11):2847–2855. doi: 10.3201/eid2711.210726 34670644 PMC8544997

[15] Cousins S. Myanmar grapples with its high tuberculosis burden. Lancet. 2017 389(10068):491–492 doi: 10.1016/S0140-6736(17)30262-3 28170324

[16] Thet Lwin ZM, Sahu SK, Owiti P, Chinnakali P, Majumdar SS. Public-private mix for tuberculosis care and control in Myanmar: a strategy to scale up? Public Health Action. 2017 7(1):15–20. doi: 10.5588/pha.16.0103 28775938 PMC5526479

[17] Nwe TT, Saw S, Le Win L, Mon MM, van Griensven J, Zhou S, et al. Engagement of public and private medical facilities in tuberculosis care in Myanmar: contributions and trends over an eight-year period Infect Dis Poverty. 2017 6(1):123. doi: 10.1186/s40249-017-0337-8 28859677 PMC5579949

[18] Lin KS, Kyaw CS, Sone YP, Win SY. Knowledge on tuberculosis among the members of a rural community in Myanmar Int J Mycobacteriol. 2017 6(3):274–280. doi: 10.4103/ijmy.ijmy_89_17 28776527

[19] Aung ST, Thu A, Aung HL, Thu M. Measuring Catastrophic Costs Due to Tuberculosis in Myanmar. Trop Med Infect Dis. 2021 6(3):130. doi: 10.3390/tropicalmed6030130 34287379 PMC8293353

