# Supplementary figures and images for: The fourth national tuberculosis prevalence survey in Myanmar
Source: PLOS Glob Public Health. 2022 Jun 14;2(6):e0000588. doi: 10.1371/journal.pgph.0000588 (PMC10021272; doi:10.1371/journal.pgph.0000588)

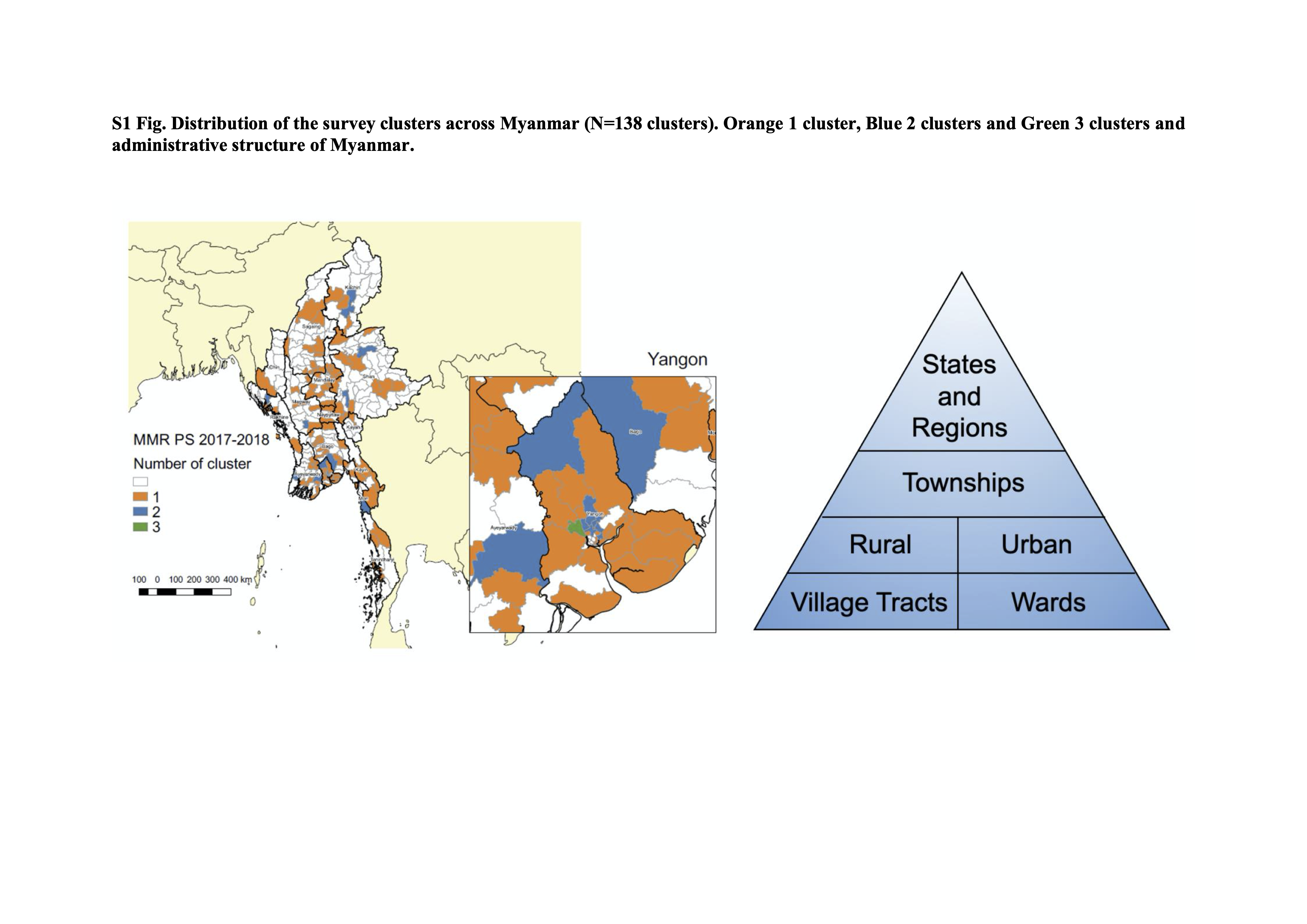

Supplement: S1 Fig — Orange 1 cluster, Blue 2 clusters and Green 3 clusters and Myanmar administrative Structure. (TIFF) [file pgph.0000588.s001.tiff]
