# Supplementary material for: The fourth national tuberculosis prevalence survey in Myanmar
Source: PLOS Glob Public Health. 2022 Jun 14;2(6):e0000588. doi: 10.1371/journal.pgph.0000588 (PMC10021272; doi:10.1371/journal.pgph.0000588)
Supplement: S1 Table — (DOCX) [file pgph.0000588.s002.docx]

S1 Table. Study participants

|  |  | National | | | | | State | | | Region | | | Yangon | | |
| --- | --- | --- | --- | --- | --- | --- | --- | --- | --- | --- | --- | --- | --- | --- | --- |
|  |  | Total eligible population | Participants | % | Nonparticipants | % | Total eligible population | Participants | % | Total eligible population | Participants | % | Total eligible population | Participants | % |
| Total |  | 75 676 | 66 480 | 88 | 9 196 | 12 | 20 832 | 18 119 | 87 | 33 824 | 30 532 | 90 | 21 020 | 17 829 | 85 |
| Sex | Female | 42 310 | 38 495 | 91 | 3 815 | 9 | 11 589 | 10 475 | 90 | 18 833 | 17 556 | 93 | 11 888 | 10 464 | 88 |
|  | Male | 33 366 | 27 985 | 84 | 5,381 | 16 | 9 243 | 7 644 | 83 | 14 991 | 12 976 | 87 | 9 132 | 7 365 | 81 |
| Age group | 15‒24 | 16 162 | 13 654 | 84 | 2 508 | 16 | 4 603 | 3 862 | 84 | 6 593 | 5 654 | 86 | 4 966 | 4 138 | 83 |
|  | 25‒34 | 15 474 | 13 151 | 85 | 2 323 | 15 | 4 076 | 3 453 | 85 | 6 830 | 6 024 | 88 | 4 568 | 3 674 | 80 |
|  | 35‒44 | 14 344 | 12 609 | 88 | 1 735 | 12 | 3 844 | 3 330 | 87 | 6 633 | 6 014 | 91 | 3 867 | 3 265 | 84 |
|  | 45‒54 | 12 761 | 11 513 | 90 | 1,248 | 10 | 3 649 | 3 259 | 89 | 5 878 | 5 463 | 93 | 3 234 | 2 791 | 86 |
|  | 55‒64 | 9 219 | 8 484 | 92 | 735 | 8 | 2 607 | 2 383 | 91 | 4 208 | 3 957 | 94 | 2 404 | 2 144 | 89 |
|  | > 65 | 7 716 | 7 069 | 92 | 647 | 8 | 2 053 | 1 832 | 89 | 3 682 | 3 420 | 93 | 1 981 | 1 817 | 92 |
| Female/ age group | 15‒24 | 8 616 | 7 549 | 88 | 1 067 | 12 | 2 398 | 2 072 | 86 | 3 483 | 3 124 | 90 | 2 735 | 2 353 | 86 |
|  | 25‒34 | 8 603 | 7 666 | 89 | 937 | 11 | 2 253 | 2 012 | 89 | 3 767 | 3 484 | 92 | 2 583 | 2 170 | 84 |
|  | 35‒44 | 8 003 | 7 366 | 92 | 637 | 8 | 2157 | 1 978 | 92 | 3 688 | 3 473 | 94 | 2 158 | 1 915 | 89 |
|  | 45‒54 | 7 206 | 6 731 | 93 | 475 | 7 | 2 061 | 1 916 | 93 | 3 314 | 3 170 | 96 | 1 831 | 1 645 | 90 |
|  | 55‒64 | 5 271 | 4 954 | 94 | 317 | 6 | 1 497 | 1 406 | 94 | 2 396 | 2 284 | 95 | 1 378 | 1 264 | 92 |
|  | > 65 | 4 611 | 4 229 | 92 | 382 | 8 | 1 223 | 1 091 | 89 | 2 185 | 2 021 | 92 | 1 203 | 1 117 | 93 |
| Male/ age group | 15‒24 | 7 546 | 6 105 | 81 | 1 441 | 19 | 2 205 | 1 790 | 81 | 3 110 | 2 530 | 81 | 2 231 | 1 785 | 80 |
|  | 25‒34 | 6 871 | 5 485 | 80 | 1 386 | 20 | 1 823 | 1 441 | 79 | 3 063 | 2 540 | 83 | 1 985 | 1 504 | 76 |
|  | 35‒44 | 6 341 | 5 243 | 83 | 1 098 | 17 | 1 687 | 1 352 | 80 | 2 945 | 2 541 | 86 | 1 709 | 1 350 | 79 |
|  | 45‒54 | 5 555 | 4 782 | 86 | 773 | 14 | 1 588 | 1 343 | 85 | 2 564 | 2 293 | 89 | 1 403 | 1 146 | 82 |
|  | 55‒64 | 3 948 | 3 530 | 89 | 418 | 11 | 1 110 | 977 | 88 | 1 812 | 1 673 | 92 | 1 026 | 880 | 86 |
|  | > 65 | 3 105 | 2 840 | 91 | 265 | 9 | 830 | 741 | 89 | 1 497 | 1 399 | 93 | 778 | 700 | 90 |
| Rural/ Urban | Rural | 46 482 | 42 343 | 91 | 4 139 | 9 | 15 021 | 13 527 | 90 | 25 615 | 23 564 | 92 | 5 846 | 5,252 | 90 |
|  | Urban | 29 194 | 24 137 | 83 | 5 057 | 17 | 5 811 | 4 592 | 79 | 8 209 | 6 968 | 85 | 15 174 | 12,577 | 83 |
