# Supplementary material for: The fourth national tuberculosis prevalence survey in Myanmar
Source: PLOS Glob Public Health. 2022 Jun 14;2(6):e0000588. doi: 10.1371/journal.pgph.0000588 (PMC10021272; doi:10.1371/journal.pgph.0000588)
Supplement: S2 Table — (DOCX) [file pgph.0000588.s003.docx]

**S2 Table. Comparison between Xpert and culture results in 70 clusters**

|  | | **Xpert result** | | | |  |
| --- | --- | --- | --- | --- | --- | --- |
|  |  | Negative | MTB | Trace | Not available | Total |
| **Culture result** | MTB | 17 | 56 | 6 | 1 | 80 |
|  | NTM | 33 | 2 | 0 | 0 | 35 |
|  | Negative | 4 362 | 143 | 106 | 130 | 4 741 |
|  | Contaminated | 87 | 1 | 3 | 2 | 93 |
|  | Not available | 97 | 3 | 2 | 37 | 139 |
|  | Total | 4 596 | 205 | 117 | 170 | 5 088 |

MTB – *Mycobacterium tuberculosis*, NTM – Non tuberculosis mycobacteria
