# Supplementary material for: The fourth national tuberculosis prevalence survey in Myanmar
Source: PLOS Glob Public Health. 2022 Jun 14;2(6):e0000588. doi: 10.1371/journal.pgph.0000588 (PMC10021272; doi:10.1371/journal.pgph.0000588)
Supplement: S3 Table — (DOCX) [file pgph.0000588.s004.docx]

**S3 Table. National prevalence rate of Xpert-positive pulmonary TB** (> **15 years old)**

| Method | TB cases | Prevalence rate per 100 000 adults (95% CI)  (multiple imputations) |
| --- | --- | --- |
| Complete case analysis | 322 | 480 (399‒561) |
| Inverse sampling probability weights | 322 | 487 (406‒569) |
| Weights and post-stratification | 322 | 468 (391‒546) |
