# Supplementary material for: The fourth national tuberculosis prevalence survey in Myanmar
Source: PLOS Glob Public Health. 2022 Jun 14;2(6):e0000588. doi: 10.1371/journal.pgph.0000588 (PMC10021272; doi:10.1371/journal.pgph.0000588)
Supplement: S4 Table — (DOCX) [file pgph.0000588.s005.docx]

**S4 Table. Prevalence-to-case notification (P/N) ratios, 2017-2018 NTPS, Myanmar^a^.**

|  | Prevalence rate per 100,000 adults | Case Notification Rate (2018) |  |
| --- | --- | --- | --- |
|  | Point estimate | Pulmonary TB | P/N ratio |
| **National** |  |  |  |
|  | 468 | 278 | 1.7 |
| **Stratum** |  |  |  |
| States | 355 | 243 | 1.5 |
| Regions other than Yangon | 485 | 233 | 2.1 |
| Yangon | 607 | 456 | 1.3 |
| **Sex** |  |  |  |
| Female | 210 | 187 | 1.1 |
| Male | 759 | 381 | 1.9 |
| **Age** |  |  |  |
| 15-24 | 156 | 143 | 1.1 |
| 25-34 | 288 | 242 | 1.2 |
| 35-44 | 507 | 284 | 1.8 |
| 45-54 | 528 | 325 | 1.6 |
| 55-64 | 872 | 409 | 2.1 |
| 65- | 1091 | 482 | 2.3 |

^a^Notification rates (2018) were obtained from the NTP database.
