# Supplementary material for: The fourth national tuberculosis prevalence survey in Myanmar
Source: PLOS Glob Public Health. 2022 Jun 14;2(6):e0000588. doi: 10.1371/journal.pgph.0000588 (PMC10021272; doi:10.1371/journal.pgph.0000588)
Supplement: S5 Table — (DOCX) [file pgph.0000588.s006.docx]

S5 Table. Preferred facility for first consultation among symptomatic participants

|  | National | | State | | Region | | Yangon | |
| --- | --- | --- | --- | --- | --- | --- | --- | --- |
|  | Number | % | Number | % | Number | % | Number | % |
| **Seeking medical care for cough lasting ≥2 weeks** | 748 | 54 | 247 | 48 | 333 | 55 | 168 | 62 |
| **Private practitioner clinic** | 246 | 33 | 72 | 29 | 102 | 31 | 72 | 43 |
| **Pharmacy** | 193 | 26 | 42 | 17 | 94 | 28 | 57 | 34 |
| **Rural/urban health center** | 151 | 20 | 67 | 27 | 70 | 21 | 14 | 8.3 |
| **Station/township hospital/TB center** | 61 | 8.2 | 25 | 10 | 29 | 8.7 | 7 | 4.2 |
| **Public hospital** | 31 | 4.1 | 15 | 6.1 | 11 | 3.3 | 5 | 3.0 |
| **Traditional healer** | 24 | 3.2 | 6 | 2.4 | 12 | 3.6 | 6 | 3.6 |
| **Private hospital** | 23 | 3.1 | 13 | 5.3 | 6 | 1.8 | 4 | 2.4 |
| **Other** | 11 | 1.5 | 3 | 1.2 | 6 | 1.8 | 2 | 1.2 |
| **Community volunteer** | 5 | 0.7 | 4 | 1.6 | 0 | 0.0 | 1 | 0.6 |
| **INGO clinic** | 3 | 0.4 | 0 | 0.0 | 3 | 0.9 | 0 | 0.0 |

INGO – International non-government organisations
