# Supplementary material for: The fourth national tuberculosis prevalence survey in Myanmar
Source: PLOS Glob Public Health. 2022 Jun 14;2(6):e0000588. doi: 10.1371/journal.pgph.0000588 (PMC10021272; doi:10.1371/journal.pgph.0000588)
Supplement: S6 Table — (DOCX) [file pgph.0000588.s007.docx]

S6 Table. Reasons for not seeking health care

|  | National | | State | | Region | | Yangon | |
| --- | --- | --- | --- | --- | --- | --- | --- | --- |
|  | Number | % | Number | % | Number | % | Number | % |
| Total | 639 |  | 267 |  | 268 |  | 104 |  |
| Not recognized as illness | 94 | 15 | 37 | 14 | 40 | 15 | 17 | 16 |
| Ignored | 261 | 41 | 79 | 30 | 118 | 44 | 64 | 62 |
| Fear of stigma | 0 | 0.0 | 0 | 0.0 | 0 | 0.0 | 0 | 0.0 |
| Self-treatment | 279 | 44 | 143 | 54 | 118 | 44 | 18 | 17 |
| Cost | 9 | 1.4 | 4 | 1.5 | 4 | 1.5 | 1 | 1.0 |
| Distance | 8 | 1.3 | 2 | 0.7 | 5 | 1.9 | 1 | 1.0 |
| Long waiting time | 3 | 0.5 | 2 | 0.7 | 1 | 0.4 | 0 | 0.0 |
| Other | 5 | 0.8 | 1 | 0.4 | 3 | 1.1 | 1 | 1.0 |

Note: The cumulative number does not add up due to multiple answers.
